# Chemical Composition, Leishmanicidal and Cytotoxic Activities of the Essential Oils from *Mangifera indica* L. var. Rosa and Espada

**DOI:** 10.1155/2014/734946

**Published:** 2014-07-20

**Authors:** Eduardo H. S. Ramos, Marcílio M. Moraes, Laís L. de A. Nerys, Silene C. Nascimento, Gardênia C. G. Militão, Regina C. B. Q. de Figueiredo, Cláudio A. G. da Câmara, Teresinha Gonçalves Silva

**Affiliations:** ^1^Departamento de Antibióticos, Universidade Federal de Pernambuco, 50670-420 Recife, PE, Brazil; ^2^Departamento de Ciências Moleculares, Universidade Federal Rural de Pernambuco, 52171-900 Recife, PE, Brazil; ^3^Departamento de Fisiologia e Farmacologia, Universidade Federal de Pernambuco, 50670-420 Recife, PE, Brazil; ^4^Departamento de Microbiologia, Centro de Pesquisa Aggeu Magalh, FIOCRUZ, 50670-420 Recife, PE, Brazil

## Abstract

The essential oils from *Mangifera indica* var. Rosa and Espada latex were obtained by hydrodistillation and analyzed using GC-FID and GC-MS. Twenty-seven components were identified. The main compound in the essential oil from *M. indica* var. Espada (EOMiE) was terpinolene (73.6%). The essential oil of *M. indica* var. Rosa (EOMiR) was characterized by high amounts of *β*-pinene (40.7%) and terpinolene (28.3%). In the test for leishmanicidal activity against promastigotes forms of *L. amazonensis*, EOMiR and EOMiE showed IC_50_ (72 h) of 39.1 and 23.0 *μ*g/mL, respectively. In macrophages, EOMiR and EOMiE showed CC_50_ of 142.84 and 158.65 *μ*g/mL, respectively. However, both were more specific to the parasite than macrophages, with values of selectivity index of 6.91 for EOMiE and 3.66 for EOMiR. The essential oils were evaluated for their cytotoxicity against the human tumor cells HEp-2, HT-29, NCI-H292, and HL-60. The EOMiR and EOMiE were most effective against the HL-60, with IC_50_ values of 12.3 and 3.6 *μ*g/mL, respectively. The results demonstrated that the essential oils of *M. indica* can destroy *L. amazonensis* and inhibit tumor cell growth. These findings contribute to the knowledge of the Brazilian biodiversity as a source of potential therapeutic agents.

## 1. Introduction

*Mangifera indica* L. is a perennial arboreal tree belonging to the family Anacardiaceae. Native to southeastern Asia, this plant had been domesticated for centuries before spreading to other parts of the tropical world. In Brazil, this tree is known commonly as “manga,” and it was first introduced into the Northeast Region in the eighteenth century by the Portugueses. Manga-Espada and manga-Rosa are the most commonly varieties cultivated in the Pernambuco state, Brazil, where their leaves are used in folk medicine to treat backaches and bronchitis [1]. Previous investigations of the biological properties of* M. indica* have found that this plant has antiviral, antibacterial, analgesic, anti-inflammatory, immunomodulatory, and antioxidant activities [2, 3]. The composition of the essential oils of* M. indica* fruits, including the Espada and Rosa varieties, has been extensively investigated [4–12], but, to our knowledge, this is the first report on the volatile components of latex from* M. indica* var. Espada and Rosa and their leishmanicidal and cytotoxicity activities.

Leishmaniasis is a disease caused by protozoan parasites of the genus* Leishmania,* a protozoa transmitted by* Phlebotomus* sp., commonly called sand flies. Leishmaniasis is one of the leading causes of morbidity and mortality worldwide and, as such, has been found to be a major global health problem, with 1.5 to 2 million humans affected by the disease annually. About 350 million people in 88 countries are estimated to be threatened by the disease [13].* Leishmania (Leishmania) amazonensis* is one of the causative agents of human cutaneous leishmaniasis in the Amazon region, Brazil, which is associated with both the simple and diffuse forms of the disease [14]. Available chemotherapy still relies on pentavalent antimonials, amphotericin B, or pentamidine. However, the use of these compounds is limited by toxicity to the host and the development of resistance by the parasites [15–18]. Hence, a search for a new active compound with potential leishmanicidal property remains essential for the development of a new antileishmanial therapy. Extracts from medicinal plants are being widely tested for leishmanicidal activity [19].

The research for new antileishmanials and anticancer drugs is imperative and has been promoted by World Health Organization, which endorses the use of traditional medicine when appropriate health services are inaccessible [20]. Moreover, due to their broad spectrum of reported biological activities [21–23], essential oils have been of interest, including leishmanicidal [24–27] and anticancer [28] activities. In addition, their hydrophobic nature makes these oils more permeable to the cell membranes, which is a very important feature for developing agents against intracellular pathogens, or act by destroying vital intracellular components of tumor cells leading to apoptosis [29]. It is also well established that antitumor drugs may also display antileishmanial activity [30].

As part of a systematic study of the chemical composition and biological properties of aromatic plants that grow in the portion of the Atlantic forest in the state of Pernambuco, the aim of the present study was to determine the chemical composition of the essential oils obtained from the latex of the fruits of* M. indica* var. Rosa and Espada, evaluate of leishmanicidal activity against promastigote forms of the* L. amazonensis,* and the cytotoxicity of these oils against HEp-2 (human larynx carcinoma), HT-29 (human colon adenocarcinoma), NCI-H292 (human lung carcinoma), and HL-60 (human leukemia) cell lines.

## 2. Materials and Methods

### 2.1. Reagents

Fetal bovine serum was purchased from Gibco (Madrid, Spain), and RPMI-1640, Dulbecco's modified Eagle medium (DMEM), dimethyl sulfoxide (DMSO), 3-(4,5-dimethylthiazol-2-yl)-2,5-diphenyltetrazolium bromide (MTT), and doxorubicin were purchased from Sigma Chemical Co. (Madrid, Spain). The monoterpenes and sesquiterpenes used to identify the components of the essential oils of the latex of fruits from* M. indica* varieties were purchased from Sigma-Aldrich Co. The monoterpenes terpinolene, *α*-pinene, *β*-pinene, and *δ*-3-carene were purchased from Sigma-Aldrich Co.

### 2.2. Plant Material

The latex of fruits of* M. indica* L. var. Rosa and Espada was collected in December 2010 on the campus of the Federal Rural University of Pernambuco (UFRPE), Recife, Pernambuco. The plants were identified by a specialist, Ladjane C. Gomes, and voucher specimens of these species were deposited in the Vasconcelos Sobrinho Herbarium-PEURF of the UFRPE under numbers 364 for* M. indica* L. var. Espada and 363 for* M. indica* L. var. Rosa.

### 2.3. Isolation of the Essential Oil from* M. indica*

The fruits were harvested with the pedicels (approximately 2 inches long) intact. Subsequently, the pedicel was detached from the fruit at the abscission zone. The fruit was immediately inverted over a glass tube, and the latex was allowed to flow into the same tube. All latex in the glass tube was stored under refrigeration at 4°C until essential oil isolation was achieved. The latex samples extracted from the two fruit varieties were treated separately. The samples were subjected to hydrodistillation for 2 h in a Clevenger-type apparatus. The oil layers were separated and dried over anhydrous sodium sulfate, stored in hermetically sealed glass containers, and refrigerated at 4°C until the chemical analysis and cytotoxicity and leishmanicidal assays occurred. The total oil yield was expressed as a percentage (g/10 g of fresh plant material).

### 2.4. Gas Chromatography and Gas Chromatography-Mass Spectrometry (GC/MS)

Quantitative GC analyses were performed with a Hewlett-Packard 5890 Series II GC apparatus equipped with a flame ionization detector (FID) by using a nonpolar DB-5 fused silica capillary column (30 m × 0.25 mm × 0.25 mm film thickness) from J & W Scientific. The oven temperature was programmed from 60 to 24°C at 3°C/min for integrating purposes. The injector and detector temperatures were set at 260°C. Hydrogen was used as a carrier gas with a 1 mL/min flow rate and a 30 psi inlet pressure. The system was operated in split mode (1 : 30). The injection volume was 0.5 *μ*L from the solution (1 : 100 of the oil in hexane). The amount of each compound was calculated from the GC peak areas in the order of elution from the DB-5 column and is expressed as a relative percentage of the total area of the chromatogram. Analyses were performed in triplicate, and the results were submitted to descriptive statistical analysis.

Qualitative GC/MS analyses were performed using a Varian GC/MS (CG: Varian 431/CG-MS: Varian 220-MS) system operating in EI mode at 70 eV. The system was operated with the same column and temperature program as those used for the GC experiments. The carrier gas was helium with a 1 mL/min flow rate, and split mode (1 : 30) was used. The injection volume was 1 *μ*L of 1 : 100-diluted oil in hexane.

The identification of the components was based on their GC retention indices (RI) with reference to a homologous series of C7-C30 n-alkanes calculated according to the Van den Dool and Kratz equation [31] by computer, matching against the mass spectral library of the GC/MS data system (NIST 98 and WILEY) and other published mass spectra [32] and by coinjection with authentic standards. Area percentages were obtained electronically from the GC-FID response without the use of an internal standard or correction factors.

### 2.5. Leishmanicidal Activity Assay on Promastigotes

*L. amazonensis* amastigotes forms (MHOM/77BR/LTB0016) were isolated from Balb/c mice lesions and maintained as promastigote forms at 26°C in Schneider's medium (Sigma-Aldrich, St. Louis, MO, USA) containing 10% inactivated fetal bovine serum (FBS) (Sigma-Aldrich, St. Louis, MO, USA), 100 *μ*g streptomycin/mL, and 100 U/mL penicillin (Sigma-Aldrich, St. Louis, MO, USA). The parasites (10^6^ parasites/mL) were incubated at 26°C in Schneider's Drosophila medium supplemented with 10% FBS in the absence, or presence, of the different concentrations of the essential oils. The cell density was determined using a Neubauer counting chamber [33]. The concentration that inhibited culture growth by 50% (IC_50_) was determined after 72 h by regression analysis using the SPSS 8.0 software. All experiments were performed in triplicate.

### 2.6. Cytotoxicity Assay on Macrophage

Peritoneal macrophages from Swiss (*Mus musculus*) mice were plated at 5 × 10^4^ cells/well in 96-well plate with RPMI medium, supplemented with 10% inactivated FBS, and incubated for 2 h at 37°C in 5% CO_2_. Nonadherent cells were then removed, and the adhered macrophages were washed twice with PBS and grown for 72 h in RPMI in the absence, or presence, of the different concentrations of EOMiR and EOMiE (100 to 6.25 *μ*g/mL). Treated and untreated cells were washed and incubated in fresh culture medium containing 5 mg/mL of 3-(4,5-dimethylthiazol-2-yl)-2,5-diphenyl tetrazolium bromide (MTT) for 3 h at 37°C under the same conditions. After incubation, the cells were solubilized in DMSO (100 *μ*L/well) and the formazan precipitates derived from MTT reduction were determined spectrophotometrically at 540 nm. The 50% cytotoxic concentration (CC_50_) was determined by regression analysis using the SPSS 8.0 for Windows. The selectivity index was determined as the ratio of CC_50_ for the macrophages to IC_50_ for the protozoa [33]. Each assay was carried out in triplicate in three independent experiments.

### 2.7. Cytotoxicity Assays on Tumoral Cell Line

The cell lines used for the cytotoxicity assays were HEp-2 (human larynx carcinoma), HT-29 (human colon adenocarcinoma), NCI-H292 (human lung mucoepidermoid carcinoma), and HL-60 (human promyelocytic leukemia), and these cells were obtained from the Cell Bank of Rio de Janeiro (Rio de Janeiro, Brazil). The HEp-2, NCI-H292, and HT-29 cells were maintained in DMEM and supplemented with 10% fetal bovine serum, 2 mM glutamine, 100 U/mL penicillin, and 100 *μ*g/mL streptomycin at 37°C with 5% CO_2_. The HL-60 cells were cultured in RPMI-1640 medium under the same conditions.

Cell counts and viability were determined by trypan blue staining. The cell concentration was adjusted to 3 × 10^5^ cells/mL for HL-60 cells and 2 × 10^5^ cells/mL for the other cell lines. Approximately 100 *μ*L of cell suspension with the above concentration was cultured in each well of 96-well plates for 24 h at 37°C in a humid atmosphere containing 5% CO_2_. After 24 h, EOMiR, EOMiE, or their major components (50.0, 25.0, 12.50, 6.25, 3.12, 1.56, or 0.78 mg/mL) “dissolved in DMSO” were added to each well of the 96-well plates, and the plates were incubated again at 37°C for 72 h. Control groups received DMSO (0.1%). Doxorubicin was used as a positive control. The growth of the tumor cells was quantified by the ability of the living cells to reduce yellow tetrazolium MTT (3-(4,5-dimethylthiazolyl-2)-2,5-diphenyltetrazolium bromide) to a blue formazan product [34]. After 72 h of incubation, MTT (5.0 mg/mL) was added to the plate. After three hours for the suspended cells or two hours for the adherent cells, the formazan product from the reduction of MTT was dissolved in DMSO, and the absorbance was measured by using a multiplate reader. The effect of the oils was quantified as the percentage of control absorbance of the reduced dye at 450 nm (Multiplate Reader Thermoplate).

## 3. Results and Discussion

### 3.1. Chemical Composition of the Essential Oils

The latex samples collected from fruits of the* M. indica* var. Espada and Rosa were viscous clear liquids with an aroma characteristic of the fresh ripe fruit. Both latex samples were submitted to hydrodistillation and yielded colorless and pleasant-smelling oils. The best yield was obtained for the EOMiR (9.50%). All constituents identified in the essential oils of the latex of the fruits of the two studied mango varieties are listed in Table 1 in the order of elution from the DB-5 column.

Twenty-seven components were identified in the oils of both varieties, representing approximately 97.0 ± 0.3% and 96.4 ± 0.5% of the EOMiE and EOMiR, respectively (Table 1). Nineteen compounds were common to both varieties. This analysis revealed that both oils were characterized by a high content of monoterpene hydrocarbons. The main compound identified in the EOMiE was terpinolene (73.6 ± 0.2%), with *δ*-3-carene (5.7 ± 0.0%) being the second most abundant. The main compound in the EOMiR was *β*-pinene (40.7 ± 0.3%), followed by terpinolene (28.3 ± 0.1%) and *α*-pinene (11.5 ± 0.1%). Both oils had similar chemical profiles, differing only in the percentages of the constituents. In fact, terpinolene, a main component identified in both oils, was found to be the major constituent of the EOMiE, but it was found at a lower percentage in the EOMiR. The same pattern was observed for *α*- and *β*-pinene, which were major compounds identified in the EOMiR, but they were detected at percentages lower than 2% in the EOMiE.

A study performed by Loveys et al. [35] reported the presence of terpinolene (83.7%) and car-3-ene (89.8%) as major components in the* Australian Kensington* and* Irwin* varieties, respectively. These compounds were also found in the present study, whereas they were the major components in only the* M. indica* var. Espada.

The chemical profile of the mango essential oils in our study, characterized by a high quantity of monoterpenes, was very similar to that reported for samples collected in different regions of the world [8, 9, 36, 37]. The main constituents found in the present study have also been identified in significant amounts in other varieties collected in other locations.

Terpinolene has been found in significant amounts in the volatile fractions of the fruits of eight varieties of mango (Chana = 62.4%, Coquinho = 51.4%, Comum = 45.4%, Carlota = 52.0%, Bacuri = 57.0%, Cheiro = 66.1%, Cametá = 56.3%, and Gorjoba = 54.8%) collected in the state of Pará, Brazil [4]. The essential oils of the pulp from the Choak Anand, Ok-rong, and yellow Keaw varieties of mango grown in Thailand contain primarily terpinolene [12]. A similar pattern was recently reported for the essential oil from the flowers of a specimen from China, which had a high content of terpinolene [37].

Contrary to the terpinolene profile found in our study, Andrade et al. [4] did not report the presence of terpinolene in the volatile fraction of* M. indica* var. Rosa and only found it in low amounts (0.2%) in the variety Espada from Pará (Brazil). According to the literature, the essential oils from the exudate of the investigated mango varieties have marked differences in composition with respect to the latex essential oils from seven varieties collected in India [36]. Specifically, these essential oils from India did not contain terpinolene, and these oils had lower contents of *β*-pinene and high contents of *β*-myrcene, limonene,* cis*-ocimene, and* trans*-ocimene.

The compound *α*-pinene was the main compound found in the oils from the fruit of the variety Qalmi [8] and from the exudate of four varieties of mango (Malgoa, Seedling, Mallika, and Totapuri) collected in India [36]. The essential oil from the leaves of a mango species from Kenya contained *α*-pinene as the main compound [5]. *β*-Pinene was found in an appreciable amount (12.5%) in a sample of fruits of the variety Qalmi collected in India [8].

### 3.2. Leishmanicidal Activity of EOMiR and EOMiE

**Figure 1:**
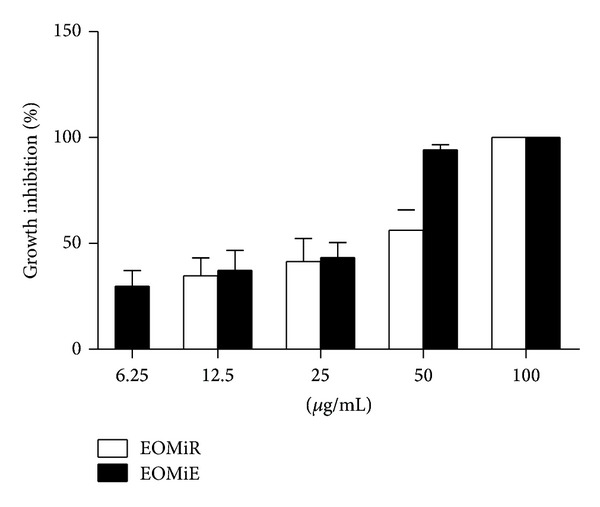
Effects of essential oils on* Leishmania amazonensis *promastigote forms. Each bar represents the mean ± standard deviation of three independent experiments in triplicate.

Our results showed that both oils demonstrated similar effect on the* L. amazonensis* promastigotes, inhibiting the parasite growth in dose-dependent manner (Figure 1). Only at a concentration of 50 *μ*g/mL did the activity profile between the two oils differ, seeing that EOMiE presented an inhibition rate of growth better than EOMiR.

The IC_50_/72 h estimated for the oils were 39.06 and 22.96 *μ*g/mL for EOMiR and EOMiE, respectively (Table 2). the oils had low cytotoxic effect on macrophages, with a CC_50_ of 142.84 and 158.65 *μ*g/mL for EOMiR and EOMiE, respectively. However, both oils were more specific to the parasite than the macrophages, with values of a selectivity index of 6.91 for EOMiE, demonstrating that it is six times more toxic to the parasite compared to macrophages.

Essential oils, as well as their components, have been found to possess a wide spectrum of pharmacological effects including antibacterial, antifungal, antiviral, antihelminthic, and antiprotozoal activities [38, 39]. They are also known to have important biological activities against trypanosomatids as* Trypanosoma brucei* [40],* Leishmania* [24], and* T. cruzi* [41]. These activities are mainly attributed to the presence of terpenic, aromatic, and aliphatic constituents [42].

It is usually assumed that terpenic constituents are responsible for the hydrophobic feature of essential oils [43] which allows essential oils to freely permeate the cell membranes and kill the parasites by affecting their cytoplasmic metabolic pathways or organelles [44]. On the other hand, essential oils themselves could interact with parasite membranes and cause drastic physiologic changes leading to the loss of membrane permeability that ultimately leads to cell death [45]. However, due to the great number of constituents and the synergistic or antagonistic interactions existing among them, it is likely that essential oils have other cellular targets besides the cellular membranes. In fact, interactions of essential oils with lipids and proteins have already been reported [45].

### 3.3. Cell Viability Assay

The cytotoxic activity of the essential oils from* M. indica* var. Rosa and Espada, and of the major terpenes in the Rosa variety (*β*-pinene, terpinolene, and *α*-pinene) and the Espada variety (terpinolene and *δ*-3-carene), was investigated against human tumor cell lines. The cytotoxic effects of the essential oils are presented in Table 3. The IC_50_ values were ranged from 12.3 to 38.9 *μ*g/mL for the EOMiR and from 3.6 to 14.5 *μ*g/mL for the EOMiE, depending upon the cell line. These values indicate that these cancer cell lines differ with respect to their sensitivity to the substances contained in the essential oil of* M. indica* latex. These differences may be due to the different molecular characteristics of these cells. Generally, the mechanism of cell death induced after treatment is dependent on several factors, such as the compounds used, their concentrations, and the cell line used for the study.

According to the criteria of the National Cancer Institute (NCI-USA) for considering a crude extract promising for further purification, the IC_50_ must be ≤30 *μ*g/mL, and, for a pure substance to be considered promising for use as an antitumor agent, it should have an IC_50_ ≤4 *μ*g/mL, which corresponds to strong cytotoxic activity [46]. The oils tested in this study had IC_50_ values below the value determined by NCI-USA for all cell lines tested. The essential oil is classified as a crude extract, and it has a complex composition, containing from a dozen to several hundred components, with terpenes being the major components.

Terpinolene and *δ*-3-carene are the major components of the EOMiE, and both were separately evaluated to determine their cytotoxic activities. Terpinolene showed cytotoxic activity against all cell lines tested but was more potent against HT-29, HEp-2, and NCI-H292 cells. *δ*-3-Carene exhibited little activity against all lines.

To evaluate the interaction between the major constituents of the essential oil, some constituents were tested separately and in mixtures with concentrations equal to those in the essential oil. When analyzed separately, terpinolene showed more potent than *δ*-3-carene, whereas when they were analyzed together [(terpinolene : *δ*-3-carene (74 : 6)] at the ratio present in the essential oil, the mixture showed lower activity than terpinolene itself. These results suggest that the cytotoxic activity displayed by the EOMiE is associated with complex interaction between the major component terpinolene and other minor components of the mixture.

The major components in the EOMiR yielded the following results when analyzed separately: *β*-pinene (IC_50_ 6.6–10.5 *μ*g/mL), terpinolene (IC_50_ 13.7–28.8 *μ*g/mL), and *α*-pinene (IC_50_ 11.0–11.8 *μ*g/mL). Both *α*- and *β*-pinene showed higher cytotoxicity than terpinolene. When a mixture of these three components was analyzed [*β*-pinene (41%) + terpinolene (28%) + *α* pinene (11%)], significant activity against all cell lines, except NCI-H292, was observed. Nevertheless, this activity appeared selective for leukemia cells because the IC_50_ value was very close to the cut-off set by the NCI for pure substances (IC_50_ ≤ 4 *μ*g/mL), despite the test substance being a mixture of terpenes.

Because strong cytotoxic activity was observed for *β*-pinene and given that it is a component of the EOMiE, the cytotoxic activity of a mixture of *β*-pinene and terpinolene was assessed. The *β*-pinene + terpinolene mixture was less active than the separate compounds and the EOMiE, indicating that there is antagonism between these two components. This result shows that the activity of the EOMiE is associated with possible synergism among all the compounds present in the oil, not just a particular component.

With respect to their biological properties, it must be kept in mind that essential oils are complex mixtures of numerous molecules, and one might wonder if their biological effects are the result of synergism among all molecules or reflect only the activities of the main molecules present at the highest concentrations according to the gas chromatographic analysis. Generally, the major components are found to reflect quite well the biophysical and biological features of the essential oils from which they were isolated, with the amplitudes of their effects being dependent on their concentration when they are tested on their own or in essential oils [47].

Because of the great number of constituents, essential oils appear to have no specific cellular targets [48], involving multiple targets at the same time [49]. As typical lipophilic compounds, these oils pass through the cell wall and the cytoplasmic membrane, disrupting the structure of the different layers of polysaccharides, fatty acids, and phospholipids and permeabilizing these structures. Cytotoxicity appears to involve such membrane damage [50].

The cytotoxicity of essential oils on mammalian cells is caused by the induction of apoptosis and necrosis. In eukaryotic cells, essential oils can induce the depolarization of the mitochondrial membranes by decreasing the membrane potential, affecting Ca^++^ and other ion channels, and reducing the pH gradient, affecting (as in bacteria) the proton pump and the ATP pool [51]. Essential oils change the fluidity of membranes, which become abnormally permeable, resulting in the leakage of radicals, cytochrome c, calcium ions, and proteins, as in the case of oxidative stress and bioenergetic failure. The permeabilization of the outer and inner mitochondrial membranes leads to cell death by apoptosis and necrosis [52]. In general, the cytotoxic activity of essential oils is primarily due to the presence of phenols, aldehydes, and alcohols [53].

## 4. Conclusions

This study revealed the chemical composition and leishmanicidal and cytotoxic activities of the essential oils of the latex of* M. indica* var. Espada and Rosa, thus contributing to the knowledge of the biological properties of the species. Little is known about the compounds of the latex of these two varieties. Monoterpenes were identified as dominant compounds in the essential oils from both varieties. Others classes of compounds, including oxygenated monoterpenes, sesquiterpene hydrocarbons, and oxygenated sesquiterpenes, were present at lower concentrations. The essential oils present a low toxicity to mammalian cell associated with significant leishmanicidal activity. The anticancer activity* in vitro *of these oils was considered promising. Further studies are required to elucidate the mechanisms of parasite death induced by the most promissory essential oils and identify their putative intracellular targets.

## Figures and Tables

**Table 1:** Chemical composition of the essential oils from the latex of two varieties of *M. indica *var. Rosa and Espada.

Compounds	RI^a^	RI^b^	*M. indica* var. Espada (%MS)	*M. indica* var. Rosa (%MS)	Method of identification
Yield (%) ± SD			5.6 ± 0.0	9.5 ± 0.1	
*α*-Pinene	930	932	0.8 ± 0.0	11.5 ± 0.1	RI, MS, CI
Camphene	941	946		0.3 ± 0.0	RI, MS, CI
Sabinene	966	969		1.9 ± 0.0	RI, MS
*β*-Pinene	979	974	1.9 ± 0.0	40.7 ± 0.3	RI, MS, CI
*δ*-2-Carene	997	1001	0.6 ± 0.0		RI, MS
*δ*-3-Carene	1005	1008	5.7 ± 0.0	2.1 ± 0.0	RI, MS
*α*-Terpinene	1012	1014	2.5 ± 0.0	1.0 ± 0.0	RI, MS, CI
*p*-Cymene	1018	1020	0.4 ± 0.0	0.3 ± 0.0	RI, MS, CI
Limonene	1023	1024	1.4 ± 0.1	1.3 ± 0.0	RI, MS, CI
Sylvestrene	1025	1025	0.9 ± 0.0	0.7 ± 0.0	RI, MS
(*E*)-*β*-Ocimene	1039	1044		0.6 ± 0.0	RI, MS
*γ*-Terpinene	1050	1054	0.4 ± 0.0	0.2 ± 0.0	RI, MS
Terpinolene	1082	1086	73.6 ± 0.2	28.3 ± 0.1	RI, MS, CI
*p*-Cymen-8-ol	1180	1179	0.7 ± 0.0	0.3 ± 0.0	RI, MS
*trans*-Chrysanthenyl acetate	1231	1235	0.3 ± 0.0		RI, MS
*cis*-Chrysanthenyl acetate	1258	1261	0.3 ± 0.0	0.3 ± 0.0	RI, MS
Isopulegyl acetate	1271	1274	0.5 ± 0.0	0.1 ± 0.0	RI, MS
*E*-Patchenol	1325	1328		0.1 ± 0.0	RI, MS
*β*-Patchoulene	1380	1379	0.3 ± 0.0	0.1 ± 0.0	RI, MS
*β*-Longipinene	1400	1400	0.8 ± 0.0	2.9 ± 0.0	RI, MS
Cycloseychellene	1407	1406	0.4 ± 0.0	0.2 ± 0.0	RI, MS
*γ*-Elemene	1435	1434	0.3 ± 0.0	1.5 ± 0.0	RI, MS, CI
Citronellyl propanoate	1442	1444		0.1 ± 0.0	RI, MS
*α*-Clovene	1449	1452	0.6 ± 0.0	0.3 ± 0.0	RI, MS
*γ*-Gurjunene	1472	1475	3.7 ± 0.2	1.3 ± 0.0	RI, MS, CI
*γ*-Muurolene	1477	1478	0.4 ± 0.0		RI, MS
*γ*-Himachalene	1477	1481	0.6 ± 0.0	0.2 ± 0.0	RI, MS
Monoterpene hydrocarbons			88.2 ± 0.2	88.9 ± 0.4	
Oxygenated monoterpenes			1.8 ± 0.1	0.8 ± 0.0	
Sesquiterpene hydrocarbons			7.1 ± 0.1	6.5 ± 0.1	
Oxygenated sesquiterpenes				0.1 ± 0.0	

Total			97.1 ± 0.3	96.3 ± 0.5	

^a^The retention indices were calculated from the retention times in relation to those of a series of n-alkanes on a 30 m DB-5 capillary column. ^b^Linear retention indices from the literature. RI: retention index; MS: mass spectrum; CI: coinjection with authentic standards.

**Table 2:** Leishmanicidal and cytotoxic effects of essentials oils of *M. indica *L.

Treatment	Promastigote IC_50_ (*μ*g/mL)	Macrophage CC_50_ (*μ*g/mL)	Selectivity index
EOMiR	39.1 ± 5.6	142.8 ± 6.0	3.7
EOMiE	23.0 ± 2.7	158.6 ± 2.5	6.9

The leishmanicidal activity was expressed as the IC_50_ and CC_50_ by the linear regression analysis, respectively. Values are mean ± standard deviation from at least three independent experiments in duplicate. Differences were considered significant at a 0.05 level of confidence. SI: CC_50_ macrophages/IC_50_ promastigote forms. IC_50_: concentration inhibiting 50% of the growth of promastigotes; CC_50_: concentration capable of causing cytotoxic effects of macrophages by 50%.

**Table 3:** Cytotoxic activity against human tumor cells of the essential oils from the *M. indica* var. Rosa and *M. indica* var. Espada.

Essential oil	^ a^IC_50_ (*µ*g/mL)			
	HT-29	HEp-2	NCI-292	HL-60
EOMiR	28.7	25.6	38.9	12.3
	20.3–30.5	20.5–31.9	31.3–48.4	9.5–15.9

EOMiE	9.2	6.2	14.5	3.6
	6.0–14.0	4.4–8.6	11.4–18.4	2.8–4.8

Terpinolene	16.7	13.7	17.4	28.8
	13.0–21.6	11.5–16.3	14.8–19.6	25.6–32.3

*α*-Pinene	10.3	11.0	11.8	11.8
	8.7–12.2	7.7–15.6	9.1–13.5	10.0–14.0

*β*-Pinene	6.6	6.3	7.5	10.5
	5.8–7.5	5.0–8.0	5.1–11.0	9.1–12.0

*δ*-3-Carene	45.5	35.0	42.6	63.1
	37.6–55.0	29.6–41.2	35.1–51.9	47.4–84.0

Terpinolene (74%) + *δ*-3-carene (6%)	22.8	26.5	29.3	27.0
	16.4–31.6	22.8–30.8	25.8–33.2	23.03–31.8

*β*-Pinene (41%) + terpinolene (28%) + *α*-pinene (11%)	22.3	30.5	40.1	7.8
	16.1–31.1	25.6–36.4	20.6–47.9	5.6–10.7

Terpinolene (74%) + *β*-pinene (2%)	39.4	35.0	30.4
	36.0–43.1	27.7–44.38	21.8–42.2

Doxorubicin	0.4	0.7	0.01	0.2
	0.3–0.5	0.2–1.4	0.004–0.3	0.01–0.2

^a^Data are presented as IC_50_ values and 95% confidence intervals obtained by nonlinear regression for all cell lines from two independent experiments. Exposure time: 72 h. HEp-2: larynx carcinoma; NCI-H292: lung mucoepidermoid carcinoma; HT-29: colon adenocarcinoma; and HL-60: promyelocytic leukemia cells. Doxorubicin was used as a positive control. EOMiR: essential oil of *M. indica *var. Rosa; EOMiE: essential oil of *M. indica *var. Espada.

## References

[1] de Albuquerque UP, de Medeiros PM, de Almeida ALS (2007). Medicinal plants of the caatinga (semi-arid) vegetation of NE Brazil: a quantitative approach. *Journal of Ethnopharmacology*.

[2] Rodeiro I, Donato MT, Jiménez N, Garrido G, Delgado R, Gómez-Lechón MJ (2007). Effects of *Mangifera indica* L. aqueous extract (Vimang) on primary culture of rat hepatocytes. *Food and Chemical Toxicology*.

[3] Pardo-Andreu GL, Barrios MF, Curti C (2008). Protective effects of *Mangifera indica* L extract (Vimang), and its major component mangiferin, on iron-induced oxidative damage to rat serum and liver. *Pharmacological Research*.

[4] Andrade EHA, Maia JGS, Zoghbi MDGB (2000). Aroma volatile constituents of Brazilian varieties of mango fruit. *Journal of Food Composition and Analysis*.

[5] Alwala OJ, Wanzala W, Inyambukho RA, Osundwa EM, Ndiege IO (2010). Characterization and evaluation of repellent effect of essential oil of *Mangifera indica* L. from Kenya. *Journal of Essential Oil-Bearing Plants*.

[6] Ansari SH, Ali M, Velasco-Negueruela A, Pérez-Alonso MJ (1999). Volatile constituents of the fruits of three mango cultivars, *Mangifera indica* L.. *Journal of Essential Oil Research*.

[7] Ansari SH, Ali M, Velasco-Negueruela A, Perez-Alonso MJ (1999). Volatile constituents of Mango (*Mangifera indica*) fruits cultivar Bombay. *Journal of Medicinal and Aromatic Plant Sciences*.

[8] Ansari SH, Ali M, Velasco-Negueruela A, Perez-Alonso MJ (2004). Characterization of volatile constituents of mango “Qalmi” (*Mangifera indica* L.) fruit. *Journal of Essential Oil Research*.

[9] Franco MRB, Rodriguez-Amaya D, Lanças FMF (2004). Compostos voláteis de três cultivares de manga (*Mangifera Indica* L.). *Food Science and Technology*.

[10] Pino JA, Mesa J, Muñoz Y, Martí MP, Marbot R (2005). Volatile components from mango (*Mangifera indica* L.) cultivars. *Journal of Agricultural and Food Chemistry*.

[11] Pino JA, Mesa J (2006). Contribution of volatile compounds to mango (*Mangifera indica* L.) aroma. *Flavour and Fragrance Journal*.

[12] Tamura H, Boonbumrung S, Yoshizawa T, Varanyanond W (2000). Volatile components of the essential oils in the pulp of four yellow
mangoes (*Mangifera indica* L.) in Thailand. *Food Science and Technology Research*.

[13] WHO (2010). Control of leishmaniasis. Report of the expert committee.

[14] Lainson R, Shaw JJ, Peters W, Killick-Kendrick E (1987). Evolution, classification and geographical distribution. *The Leishmaniases in Biologyand Medicine*.

[15] Davis AJ, Murray HW, Handman E (2004). Drugs against leishmaniasis: a synergy of technology and partnerships. *Trends in Parasitology*.

[16] Goto H, Lindoso JAL (2010). Current diagnosis and treatment of cutaneous and mucocutaneous leishmaniasis. *Expert Review of Anti-Infective Therapy*.

[17] Polonio T, Efferth T (2008). Leishmaniasis: drug resistance and natural products (review). *International Journal of Molecular Medicine*.

[18] Berman J (2003). Current treatment approaches to leishmaniasis. *Current Opinion in Infectious Diseases*.

[19] Sen R, Chatterjee M (2011). Plant derived therapeutics for the treatment of Leishmaniasis. *Phytomedicine*.

[20] Rocha LG, Almeida JRGS, Macêdo RO, Barbosa-Filho JM (2005). A review of natural products with antileishmanial activity. *Phytomedicine*.

[21] Rhayour K, Bouchikhi T, Tantaoui-Elaraki A, Sendide K, Remmal A (2003). The mechanism of bactericidal action of oregano and clove essential oils and of their phenolic major components on *Escherichia coli* and *Bacillus subtilis*. *Journal of Essential Oil Research*.

[22] Kulevanova S, Kaftandzieva A, Dimitrovska A, Stefkov G, Grdanoska T, Panovski N (2000). Investigation of antimicrobial activity of essential oils of several Macedonian *Thymus* L. species (Lamiaceae). *Bollettino Chimico Farmaceutico*.

[23] Marino M, Bersani C, Comi G (1999). Antimicrobial activity of the essential oils of *Thymus vulgaris* L. measured using a bioimpedometric method. *Journal of Food Protection*.

[24] de Medeiros MDGF, da Silva AC, Citó AMDGL (2011). In vitro antileishmanial activity and cytotoxicity of essential oil from *Lippia sidoides* Cham. *Parasitology International*.

[25] Rosa MDSS, Mendonça-Filho RR, Bizzo HR (2003). Antileishmanial activity of a linalool-rich essential oil from *Croton cajucara*. *Antimicrobial Agents and Chemotherapy*.

[26] Zheljazkov VD, Cantrell CL, Tekwani B, Khan SI (2007). Content, composition, and bioactivity of the essential oils of three basil genotypes as a function of harvesting. *Journal of Agricultural and Food Chemistry*.

[27] Mikus J, Harkenthal M, Steverding D, Reichling J (2000). In vitro effect of essential oils and isolated mono- and sesquiterpenes on *Leishmania major* and *Trypanosoma brucei*. *Planta Medica*.

[28] Mothana RA, Al-Said MS, Al-Yahya MA, Al-Rehaily AJ, Khaled JM (2013). GC and GC/MS analysis of essential oil composition of the endemic Soqotraen Leucas virgata Balf.f. and its antimicrobial and antioxidant activities. *International Journal of Molecular Sciences*.

[29] Sanchez-Suarez JF, Riveros I, Delgado G (2013). Evaluation of the leishmanicidal and cytotoxic potential of essential oils derived from ten Colombian plants. *Iranian Journal of Parasitology*.

[30] Fuertes MA, Nguewa PA, Castilla J, Alonso C, Pérez JM (2008). Anticancer compounds as leishmanicidal drugs: challenges in Chemotherapy and future perspectives. *Current Medicinal Chemistry*.

[31] van den Dool H, Dec. Kratz P (1963). A generalization of the retention index system including linear temperature programmed gas-liquid partition chromatography. *Journal of Chromatography A*.

[32] Sparkman OD (2005). Identification of essential oil components by gas chromatography/quadrupole mass spectroscopy Robert P. Adams. *Journal of the American Society for Mass Spectrometry*.

[33] de Medeiros MDGF, da Silva AC, Citó AMDGL (2011). *In vitro* antileishmanial activity and cytotoxicity of essential oil from Lippia sidoides Cham. *Parasitology International*.

[34] Mosmann T (1983). Rapid colorimetric assay for cellular growth and survival: application to proliferation and cytotoxicity assays. *Journal of Immunological Methods*.

[35] Loveys BR, Robinson SP, Brophy JJ, Chacko EK (1992). Mango Sapburn: components of fruit sap and their role in causing skin damage. *Australian Journal of Plant Physiology*.

[36] Saby John K, Jagan Mohan Rao L, Bhat SG, Prasada Rao UJS (1999). Characterization of aroma components of sap from different Indian mango varieties. *Phytochemistry*.

[37] Wang HW, Liu YQ, Wei SL, Yan ZJ, Lu K (2010). Comparison of microwave-assisted and conventional hydrodistillation in the extraction of essential oils from mango (*Mangifera indica* L.) flowers. *Molecules*.

[38] Macedo ITF, Bevilaqua CML, de Oliveira LMB (2010). Anthelmintic effect of Eucalyptus staigeriana essential oil against goat gastrointestinal nematodes. *Veterinary Parasitology*.

[39] Santos AO, Santin AC, Yamaguchi MU (2010). Antileishmanial activity of an essential oil from the leaves and flowers of *Achillea millefolium*. *Annals of Tropical Medicine and Parasitology*.

[40] Otoguro K, Iwatsuki M, Ishiyama A (2011). In vitro antitrypanosomal activity of plant terpenes against *Trypanosoma brucei*. *Phytochemistry*.

[41] Santoro GF, Cardoso MG, Guimarães LGL, Freire JM, Soares MJ (2007). Anti-proliferative effect of the essential oil of *Cymbopogon citratus* (DC) Stapf (lemongrass) on intracellular amastigotes, bloodstream trypomastigotes and culture epimastigotes of *Trypanosoma cruzi* (Protozoa: Kinetoplastida). *Parasitology*.

[42] Schelz Z, Hohmann J, Molnar J, Chattopadhyay D (2010). Recent advances in research of antimicrobial effects of essential oils and plant derived compounds on bacteria. *Ethnomedicine: A Source of Complementary Therapeutics*.

[43] Burt SA, Vlielander R, Haagsman HP, Veldhuizen EJA (2005). Increase in activity of essential oil components carvacrol and thymol against Escherichia coli O157:H7 by addition of food stabilizers. *Journal of Food Protection*.

[44] Knobloch K, Pauli A, Iberl B, Weigand H, Weis N (1989). Antibacterial and antifungal properties of essential oil componentes. *Journal of Essential Oil Research*.

[45] Bakkali F, Averbeck S, Averbeck D, Idaomar M (2008). Biological effects of essential oils—a review. *Food and Chemical Toxicology*.

[46] Suffness M, Pezzuto JM, Hostettmann K (1991). Assays for bioactivity. *Methods in Plant Biochemistry*.

[47] Ipek E, Zeytinoglu H, Okay S, Tuylu BA, Kurkcuoglu M, Baser KHC (2005). Genotoxicity and antigenotoxicity of Origanum oil and carvacrol evaluated by Ames Salmonella/microsomal test. *Food Chemistry*.

[48] Carson CF, Mee BJ, Riley TV (2002). Mechanism of action of *Melaleuca alternifolia* (tea tree) oil on *Staphylococcus aureus*determined by time-kill, lysis, leakage, and salt tolerance assays and electron microscopy. *Antimicrobial Agents and Chemotherapy*.

[49] di Pasqua R, Hoskins N, Betts G, Mauriello G (2006). Changes in membrane fatty acids composition of microbial cells induced by addiction of thymol, carvacrol, limonene, cinnamaldehyde, and eugenol in the growing media. *Journal of Agricultural and Food Chemistry*.

[50] Sikkema J, de Bont JAM, Poolman B (1995). Mechanisms of membrane toxicity of hydrocarbons. *Microbiological Reviews*.

[51] Vercesi AE, Kowaltowski AJ, Grijalba MT, Meinicke AR, Castilho RF (1997). The role of reactive oxygen species in mitochondrial permeability transition. *Bioscience Reports*.

[52] Armstrong JS (2006). Mitochondrial membrane permeabilization: the sine qua non for cell death. *BioEssays*.

[53] Sacchetti G, Maietti S, Muzzoli M (2005). Comparative evaluation of 11 essential oils of different origin as functional antioxidants, antiradicals and antimicrobials in foods. *Food Chemistry*.

